# Mucosal Vaccination with a Newcastle Disease Virus-Vectored Vaccine Reduces Viral Loads in SARS-CoV-2-Infected Cynomolgus Macaques

**DOI:** 10.3390/vaccines12040404

**Published:** 2024-04-10

**Authors:** Bryce M. Warner, Mable Chan, Nikesh Tailor, Robert Vendramelli, Jonathan Audet, Courtney Meilleur, Thang Truong, Lauren Garnett, Marnie Willman, Geoff Soule, Kevin Tierney, Alixandra Albietz, Estella Moffat, Rick Higgins, Lisa A. Santry, Alexander Leacy, Phuc H. Pham, Jacob G. E. Yates, Yanlong Pei, David Safronetz, James E. Strong, Leonardo Susta, Carissa Embury-Hyatt, Sarah K. Wootton, Darwyn Kobasa

**Affiliations:** 1 Special Pathogens, National Microbiology Laboratory, Public Health Agency of Canada, Winnipeg, MB R3E 3R2, Canada; mable.hagan@phac-aspc.gc.ca (M.C.); nikesh.tailor@phac-aspc.gc.ca (N.T.); robert.vendramelli@phac-aspc.gc.ca (R.V.); jonathan.audet@phac-aspc.gc.ca (J.A.); courtney.meilleur@phac-aspc.gc.ca (C.M.); thang.truong@phac-aspc.gc.ca (T.T.); lauren.garnett@phac-aspc.gc.ca (L.G.); marnie.hustins@phac-aspc.gc.ca (M.W.); geoff.soule@phac-aspc.gc.ca (G.S.); kevin.tierney@phac-aspc.gc.ca (K.T.); alix.albietz@inspection.gc.ca (A.A.); david.safronetz@phac-aspc.gc.ca (D.S.); jim.strong@phac-aspc.gc.ca (J.E.S.); darwyn.kobasa@phac-aspc.gc.ca (D.K.); 2 Department of Medical Microbiology and Infectious Diseases, University of Manitoba, Winnipeg, MB R3E 0J9, Canada; 3 National Centre for Foreign Animal Disease, Canadian Food Inspection Agency, Winnipeg, MB R3E 3R2, Canada; estella.moffat@inspection.gc.ca (E.M.); carissa.emburyhyatt@inspection.gc.ca (C.E.-H.); 4 Department of Radiology, Health Sciences Center, Winnipeg, MB R3A 1S1, Canada; rhiggins@hsc.mb.ca; 5 Department of Pathobiology, University of Guelph, Guelph, ON N1G 2W1, Canada; lisa.santry@gmail.com (L.A.S.); aleacy@uoguelph.ca (A.L.); phpham@uoguelph.ca (P.H.P.); jyates01@uoguelph.ca (J.G.E.Y.); ypei@ovc.uoguelph.ca (Y.P.); lsusta@uoguelph.ca (L.S.);

**Keywords:** Newcastle disease virus, SARS-CoV-2, COVID-19, vaccine, cynomolgus macaque

## Abstract

Severe acute respiratory syndrome coronavirus 2 (SARS-CoV-2) emerged following an outbreak of unexplained viral illness in China in late 2019. Since then, it has spread globally causing a pandemic that has resulted in millions of deaths and has had enormous economic and social consequences. The emergence of SARS-CoV-2 saw the rapid and widespread development of a number of vaccine candidates worldwide, and this never-before-seen pace of vaccine development led to several candidates progressing immediately through clinical trials. Many countries have now approved vaccines for emergency use, with large-scale vaccination programs ongoing. Despite these successes, there remains a need for ongoing pre-clinical and clinical development of vaccine candidates against SARS-CoV-2, as well as vaccines that can elicit strong mucosal immune responses. Here, we report on the efficacy of a Newcastle disease virus-vectored vaccine candidate expressing SARS-CoV-2 spike protein (NDV-FLS) administered to cynomolgus macaques. Macaques given two doses of the vaccine via respiratory immunization developed robust immune responses and had reduced viral RNA levels in nasal swabs and in the lower airway. Our data indicate that NDV-FLS administered mucosally provides significant protection against SARS-CoV-2 infection, resulting in reduced viral burden and disease manifestation, and should be considered as a viable candidate for clinical development.

## 1. Introduction

In late 2019, the novel coronavirus, severe acute respiratory syndrome coronavirus 2 (SARS-CoV-2), emerged, causing an outbreak of respiratory disease named coronavirus disease 2019 (COVID-19), which was officially declared a pandemic in March of 2020 [1,2]. Since then, there have been more than 700 million recorded cases of COVID-19 and 6.5 million deaths worldwide, and the documented case and fatality numbers may be underestimated [3,4]. Most COVID-19 cases range from asymptomatic to mild–moderate severity, although severe cases (estimated to be up to 5% of all cases, varying by age and other factors) with associated acute respiratory distress syndrome and vascular complications can result in the need for mechanical ventilation [5,6]. Despite early large-scale efforts to develop and test a wide range of therapeutics, there remains no standard or broadly effective treatment option, though significant progress has been made and certain antiviral options do exist [7]. The spread of SARS-CoV-2 from person-to-person occurs mainly through close contact, inhalation of respiratory droplets or aerosols, and through fomites [8,9]. Viral transmission can occur while those infected are either asymptomatic or presymptomatic, making it difficult to identify contagious individuals and exacerbating community spread of the virus [10,11]. Additionally, it appears that the immunity granted by current vaccines wanes after a few months, allowing for vaccinated people to become infected and transmit the virus, although vaccinated people present with much milder clinical pictures compared to non-vaccinated people [12,13]. This is exacerbated by continually evolving variants of concerns (VOCs; mainly descendants of Omicron lineages), against which antibodies induced by first generation vaccines show decreased neutralizing activity [14,15,16,17,18]. Updated mRNA vaccines have helped in this regard, and continual monitoring of circulating variants and updated vaccines will likely occur for the foreseeable future [19]. Waning immunity and viral evolution make preventing transmission difficult. Currently, widespread vaccination, including mucosal vaccination, is likely the best option to reduce community spread of SARS-CoV-2.

The emergence of SARS-CoV-2 has resulted in an unprecedented global scientific response that has included the rapid development of novel vaccines and therapeutics [20]. Several vaccine candidates, specifically targeting the viral spike protein, were developed with a massive effort to complete phase III efficacy trials in record time [21,22,23,24,25]. A number of vaccines have been used in different countries. However, the limitations of waning immunity and continued viral evolution have stressed the importance of developing new next-generation vaccines, which can promote respiratory mucosal immunity and afford protection against clinical signs as well as significantly decreasing virus spread [26,27,28]. Therefore, there remains a need to develop and test alternative vaccines against SARS-CoV-2 in the context of the current pandemic as well as possible future pandemics.

We previously reported that a recombinant Newcastle disease virus (NDV)-vectored vaccine provides significant protection in a Syrian hamster model of SARS-CoV-2 infection [29]. An NDV-based vaccine expressing a full-length SARS-CoV-2 spike protein (NDV-FLS) was able to provide protection against weight loss and eliminate viral replication in the upper and lower airway when given as a single dose or in a prime-boost regimen. NDV-vectored vaccines offer several advantages including an excellent safety profile, an absence of pre-existing immunity in humans, an easily manipulatable genome, and immunogenicity when administered via different routes, including through the respiratory mucosa, which may provide an added advantage of limiting viral replication in the upper airway, reducing virus spread [29,30,31,32,33,34,35]. Several other NDV-based vaccine candidates have been developed and described, with some inactivated and live-attenuated candidates advancing into human trials [36,37,38,39,40,41,42,43]. Here, we vaccinated cynomolgus macaques with NDV-FLS to assess the vaccine’s efficacy in a non-human primate model of SARS-CoV-2 infection. Prime-boost vaccination was safe and immunogenic when administered via an intranasal and intratracheal route, with the goal of inducing upper airway and pulmonary immunity. Upon challenge with SARS-CoV-2, the vaccine was able to significantly reduce viral RNA levels in the upper airway and the lungs of infected macaques. Our data indicate that vaccination with an NDV-vectored vaccine can provide protection from infection and disease in non-human primates and provides a safe and viable option for protection against COVID-19.

## 2. Materials and Methods

### 2.1. Ethics Statement

The experiments described were carried out at the National Microbiology Laboratory of the Public Health Agency of Canada. Experiments were approved by the institutional Animal Care Committee of the Canadian Science Centre for Human and Animal Health (CSCHAH), according to the guidelines of the Canadian Council on Animal Care, in Animal User Document H-20-007. The study is reported in accordance with ARRIVE guidelines.

### 2.2. Cells and Viruses

SARS-CoV-2 (Canada/ON-VIDO-01/2020; EPI_ISL_425177) was isolated from a positive patient sample. Stocks of virus were grown in VeroE6 cells. SARS-CoV-2 virus stocks were titered by plaque assay before being used for subsequent in vivo experiments. All viruses used for in vivo experiments were from passage two. NDV expressing full-length SARS-CoV-2 spike protein (NDV-FLS) used for vaccination was obtained and grown as previously described (29). Briefly, the full-length cDNA genome of lentogenic NDV LaSota strain was designed based on Genbank accession AF077761.1 to contain a GFP reporter gene and essential NDV-specific RNA transcriptional signals, flanked by a 5′ XbaI site and a 3′ MluI site at nucleotide position 3143 between the P and M genes. A leucine to alanine mutation at position 289 was also introduced into the fusion gene. The full-length recombinant clone was synthesized de novo using a synthesis service (GeneArt, ThermoFisher, Waltham, MA, USA). To construct recombinant NDV expressing SARS-CoV-2 Spike, forward 5′GCACCGAGTTCCCCCTCTAGATTAGAAAAAATACGGGTAGAACCGC CAC-3′ and reverse 5′GTTGGACCTTGGGTACGCGTTTATCAGGTGTAGTGCAGCTTCAC-3′ primers were used to amplify human codon optimized SARS-CoV-2 full-length spike. Infusion cloning was used to insert transgenes into the NDV backbone according to the manufacturer’s protocol (Takara Bio, San Jose, CA, USA), with the 5′ end of the primer spanning 15 bp of homology with each end of the linearized vector including the XbaI or MluI sites. Viruses were rescued from cDNA and recombinant virus identity confirmed by RT-PCR and sequencing. Vero and VeroE6 cells (ATCC, Manassas, VA, USA) used for all experiments were maintained in Minimal Essential Medium (MEM) (Hyclone, Logan, UT, USA) supplemented with 5% Bovine Growth Serum Supplemented Calf (Hyclone) and L-glutamine (Gibco, Waltham, MA, USA). 293T-ACE2 cells were maintained in Dulbecco’s modified Eagle’s medium (DMEM), with 10% heat-inactivated Fetal Bovine Serum (FBS), 2 mM L-glutamine, 100 U/mL penicillin, and 100 µg/mL streptomycin. Cells were kept at 37 °C with 5% CO_2_.

### 2.3. Experimental Design

Animals were housed in containment level 4 (CL-4) for all procedures described and all infectious work was performed under CL-4 conditions. All efforts were made to minimize animal suffering and to reduce the number of animals used. All procedures described were carried out under ketamine anesthesia (10 mg/kg) given by intramuscular injection, followed by maintenance with inhalation of 3–5% isoflurane in oxygen. Animals were provided water *ad libitum* and were monitored twice daily for signs of illness or distress. Animals were fed a diet consisting of primate chow (LabDiet) daily, as well as fresh fruits and vegetables.

Eight cynomolgus macaques (*Macaca fascicularis*) (four male, four female), weighing between 2 and 3 kg, were previously used to test the efficacy of an anti-Sudan virus therapeutic and survived Sudan virus challenge. This initial studied consisted of a 28-day period following infection that involved daily monitoring and periodic sampling. Following the 28 days, each of the animals remained inside CL-4 and were monitored for an additional 28 days and allowed to recover from Sudan virus infection. Following this 28-day interval, all animals were sampled and hematological and biochemical parameters were examined along with blood immunophenotyping to determine if any residual effects remained from the initial Sudan virus challenge. All parameters that were examined had returned to within baseline range, and upon examination, all animals were determined to be healthy enough to be included in our current study. Additionally, RT-qPCR was run on all tissue samples upon necropsy to determine if residual or persistent SUDV was present in the animals. All samples tested were negative for SUDV RNA (data not shown).

**Figure 1:**
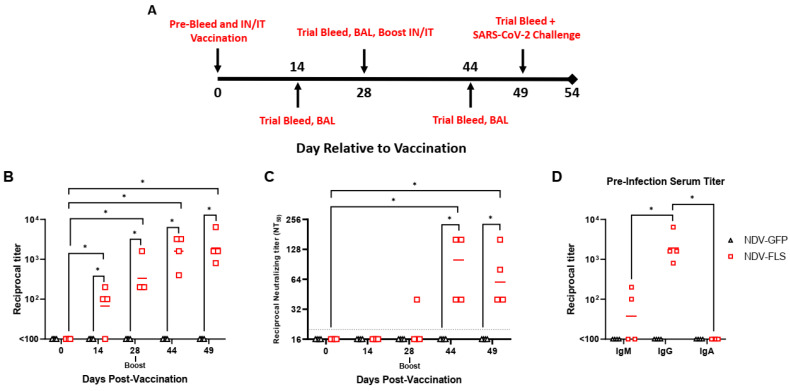
Immunogenicity of NDV expressing SARS-CoV-2 spike protein in cynomolgus macaques. A schematic representing the course of the experiment and experimental collection days is shown in (**A**). All animals were also swabbed and vital signs taken on each day pi. SARS-CoV-2-specific IgG titers (**B**) and neutralization titers (**C**) in vaccinated animals following the first and second vaccine doses. SARS-CoV-2-specific IgM, IgG, and IgA titers in animals on the day of infection shown in (**D**). Day 49 IgG titers shown in (**B**) are the same data as shown in (**D**). Significance was assessed by Mann–Whitney test. * *p* < 0.05.

From that point, all eight animals were randomly assigned to either the vaccine or control group and animals were vaccinated intranasally (1 mL) and intratracheally (1 mL) with 2.5 × 10^7^ PFU/mL each of either NDV-FLS or NDV-GFP. Following vaccination, animals were sampled periodically according to the schedule outlined in Figure 1A. Each time point consisted of a trial bleed from the femoral vein for collection of whole blood, serum, and plasma, as well as oral, nasal, rectal swabs, and X-rays (Figure 1A). Bronchoalveolar lavage (BAL) was collected at specific time points for detection of specific immune cell types, antibodies, cytokines, and chemokines and SARS-CoV-2 RNA. For SARS-CoV-2 infection, each animal was infected intratracheally (4 mL), intranasally (1 mL; 0.5 mL per nare), intraocularly (0.5 mL; 0.25 mL per eye), and orally (1.5 mL) with 1.43 × 10^5^ PFU/mL of SARS-CoV-2 for a total inoculum of 1 × 10^6^ PFU. For euthanasia on day five pi, all animals were sampled as previous and blood was collected via cardiac puncture followed by intracardiac injection of pentobarbital (100 mg/kg). For X-rays, anaesthetized animals were placed on the table and X-rays were taken at 0.8 mA at 58 KvP, with a distance of 87 cm on an X-ray plate (Konica Minolta). All X-rays were taken in V/D position (Ventral/Dorsal) and Left Lateral positions. The X-ray source was from Soyee Products Inc. (New York, NY, USA), and images were obtained and analysed using Konica Minolta Image Pilot Software version 1.80R04E.

### 2.4. Bronchoalveolar Lavage

Anaesthetized animals were placed in the prone position and were intubated intratracheally. Following intubation, animals were placed in left lateral recumbency, and a sterile tracheal suction catheter was inserted into the endotracheal tube through the trachea and into the bronchi. An injection of 8–11 mL of sterile phosphate-buffered saline (PBS) was infused into the catheter, followed by aspiration and collection of the infused PBS. The same procedure was repeated two–three times. A pulse oximeter was used throughout the procedure to monitor the animal’s heart rate and oxygen saturation and the animal was provided supplemental oxygen. BAL fluid (BALF) was spun down at 600× *g* for 10 min to separate cells and BALF. Cells were then used for downstream flowcytometry and BALF for SARS-CoV-2 detection. BAL samples were collected prior to vaccination, on days 14, 28, and 44 after vaccination and on days 2 and 5 post-challenge with SARS-CoV-2.

### 2.5. Blood Hematology, Biochemistry, and Coagulation

Complete blood counts (CBCs) and hematological analysis was obtained using a VetScan HM5 (Abaxis Veterinary Diagnostics, California City, CA, USA), as per manufacturer’s instructions. Serum biochemistry values were determined with a VetScan VS2 (Abaxis Veterinary Diagnostics) using complete diagnostic profile disks according to manufacturer’s instructions. Coagulation was assessed using a STart4 hemostasis analyzer (Diagnostica Stago, Asnières sur Seine, France) per manufacturer instructions.

### 2.6. Detection and Quantification of SARS-CoV-2 in Samples

For measurement of viral titers in swabs and tissues, tissue culture infectious dose 50 (TCID50) assays were performed. Samples were frozen at −80 °C for storage and then were thawed. In the case of tissues, each was placed in MEM supplemented with 1× L-glutamine and 1% FBS and homogenized with 5 mm stainless steel beads in a Bead Ruptor Elite Tissue Homogenizer (Omni) for 1 min at a frequency of 4-1. Homogenates were clarified by centrifugation at 1500× *g* for 10 min and ten-fold serial dilutions of tissue homogenates were made in MEM supplemented with 1× L-glutamine, 1% FBS, and a 2× dose of penicillin/streptomycin. For swabs, each swab was performed and stored in the same medium as above. For TCID50 assays, 10-fold dilutions of swab media or tissue homogenates were added to 90–100% confluent Vero cells in triplicate wells and cytopathic effect (CPE; determined by the presence of syncytia, cell rounding, and/or cell detachment as compared with uninfected cells) was read on day 5 pi. TCID50 values per mL or gram of tissue were calculated using the Reed and Muench method [44].

For detection of viral RNA, tissues collected were stored in RNAlater. Tissue RNA was extracted using an RNeasy mini plus kit (Qiagen, Hilden, Germany), according to manufacturer’s instructions. For viral RNA present in blood and swabs, RNA was extracted using a viral RNA mini kit (Qiagen). Detection of SARS-CoV-2 by RT-qPCR was performed on a QuantStudio 5 instrument (Applied Biosystems, Foster City, CA, USA) using a TaqPath 1-step RT-qPCR Master Mix (Applied Biosystems) and primers specific for the E gene of SARS-CoV-2 as per the diagnostic protocol recommended by the World Health Organization (forward—ACAGGTACGTTAATAGTTAATAGCGT; reverse—ATATTGCAGCAGTACGCACACA; Probe—FAM-ACACTAGCCATCCTTACTGCGCTTCGBBQ). Oligonucleotide concentrations were 400 nM. Stages were as follows: UNG incubation (25 °C for 2 min), reverse transcription (53 °C for 10 min), polymerase activation (95 °C for 2 min), followed by amplification (40 cycles of 95 °C for 3 s and 60 °C for 30 s). Viral E gene copy numbers were calculated using a standard curve of synthesized DNA included on each RT-qPCR plate.

### 2.7. Serological Analysis

Ninety-six-well half area flat-bottom high-binding microplates (Corning, New York, NY, USA) were coated with 30 ng/well of recombinant SARS-CoV-2 spike protein in PBS overnight at 4 °C. The next day, plates were washed four times with PBS-T (PBS + 0.1% Tween 20) and then blocked with blocking buffer (PBS-T + 5% skim milk powder) for 1 h at 37 °C. Following blocking, plates were washed four times with PBS-T and serum samples from vaccinated animals were serially diluted and added to the plates in triplicate for 1 h at 37 °C. Plates were then washed with PBS-T four times and secondary peroxidase-labelled anti-monkey IgG (KPL) was added to the plates at a dilution of 1:1000 for 1 h at 37 °C. Plates were once again washed with PBS-T and ABTS substrate was added to the plates for 30 min at room temperature. The plate reaction was stopped by adding 100 µL of 1 M H_2_SO_4_ and then OD405nm readings were taken. Positive samples were those with and absorbance greater than the absorbance of the mean of negative control wells + 3 standard deviations.

For determination of anti-SARS-CoV-2 neutralizing antibody titers, a microneutralization assay using infectious SARS-CoV-2 was used. Vero cells were seeded into 96-well plates in 100 μL medium and cultured overnight at 37 °C and 5% CO_2_. Twenty-four hours later, two-fold dilutions of heat-inactivated NHP sera starting at a 1:10 dilution were made and mixed with 400 plaque-forming units of SARS-CoV-2 at a 1:1 ratio. Virus/sera mixtures were incubated for 1 h at 37 °C. Media was removed from the cells and 100 μL of virus/sera mixtures was added to the cells in triplicate wells and incubated for 3–5 days at 37 °C and 5% CO_2_. Plates were scored for CPE and neutralization titers were calculated as the dilution of sera that resulted in the prevention of CPE in >50% of wells at that dilution. Reported are the reciprocal dilutions at which this prevention occurred.

### 2.8. Immunophenotyping via Flowcytometry

BAL cell samples were incubated for 10 min at room temperature with Human TruStain FcX and Ghost Dye Red 780 viability dye (Tonbo Biosciences, San Diego, CA, USA) before staining with CD4 PerCP-Cy5.5 (clone L200, BD Biosciences, Mississauga, ON, Canada), CD45RA PE-CF594 (clone 5H9, BD Biosciences), CD3 Alexa Fluor 700 (clone SP34-2, BD Biosciences), CCR7 BV711 (clone G043H7, BioLegend, San Diego, CA, USA), CD8 BV786 (clone RPA-T8, BD Biosciences), CD45 BUV395 (clone D058-1283, BD Biosciences). Erythrocytes were removed via incubation with 1× BD Biosciences FACS Lysing solution before fixation and permeabilization of the remaining cells with BD Biosciences Cytofix/Cytoperm solution and removal from CL-4. Following removal from containment, all samples were examined using a BD Biosciences FACSymphony A5 flow cytometer; fluorescence spillover was corrected for using singly stained compensation beads (Invitrogen UltraComp eBeads or ArC Amine Reactive Beads). Data were analyzed using FlowJo version 10 software.

### 2.9. Histopathological Analysis

Tissues were fixed in 10% neutral phosphate-buffered formalin, routinely processed, sectioned at 5 µm, and stained with hematoxylin and eosin (HE) for histopathologic examination. Semi-quantitative lesion scoring was performed as follows: The percentage affected of each section examined was scored as 0 = no pathological changes, 1 = ≤25% of lung section affected, 2 = >25% and ≤50% of lung section affected, 3 = >50% and ≤75% of lung section affected, and 4 = >75% of lung section affected. An average score of all sections for each animal was assigned out of a total score of 12. Additionally, a score of 0 (not present) or 1 (present) was given for each of the following nominal parameters, as observed in any of the sampled lobes: bronchiolitis (defined as inter-epithelial inflammatory cells, necrosis of bronchiolar epithelium and debris in lumen), diffuse alveolar damage (defined as necrosis of alveolar epithelial cells, cellular debris in alveoli and intra-alveolar fibrin), alveolar edema, alveolar hemorrhage, interstitial pneumonia nodular, hyperplasia of type II pneumocytes, perivasculitis and peribronchiolitis. For each animal, a score out of 8 was given, and group averages were compared using a 2-sample *t*-test.

### 2.10. Immunohistochemistry

Paraffin tissue sections were quenched for 10 min in aqueous 3% hydrogen peroxide. Epitopes were retrieved using an in-house glycan retrieval solution, in a Biocare Medical Decloaking Chamber. The primary antibody applied to the sections was SARS-CoV-2 (2019-nCoV) Nucleocapsid, Rabbit MAb (Sino Biological Inc. #40143-R019, Beijing, China). It was used at a 1:6000 dilution for thirty minutes. The sections were then visualized using a horse radish peroxidase-labelled polymer, Envision^®^ + system (anti-rabbit) (Dako, Agilent, Santa Clara, CA, USA), and reacted with the chromogen diaminobenzidine (DAB). The sections were then counter-stained with Gill’s hematoxylin.

### 2.11. In Situ Hybridization

For the ISH technique, 5 um paraffin-embedded formalin-fixed tissue sections were cut, air-dried, and then melted on to the charged slides in a 600 °C oven. Then, the slides were cleared and hydrated in xylene and 100% ethanol then air dried. The sections were quenched for 10 min in aqueous H_2_O_2_, boiled in target retrieval solution for 15 min, rinsed in 100% ethanol and air-dried again. Then, a final pre-treatment of protease plus enzyme for 15 min at 400 °C was applied. The probe (V-nCoV2019-S ref#848561, from Advanced Cell Diagnostics, Newark, CA, USA) was applied and incubated at 40 °C for 2 h. Then, the hybridization amplification steps (AMP 1-6) were applied to the slides for the recommended times and temps as per the manual and as per manufacturer instructions (RNAscope^®^ 2.5HD Detection Reagent–Red kit (ACD)). The signal was then visualized by the chromogen Fast Red. The sections were then counter stained with Gill’s 1 hematoxylin, dried, and read.

### 2.12. Luminex Multiplex Assay

Cytokine responses in BALF samples were examined using the Cytokine Monkey Magnetic 29-Plex Panel for Luminex Platform (ThermoFisher Scientific). Cytokines that were analysed included: G-CSF, IFN-γ, IL-10, IL-12, IL-17A, IL-2, IL-4, IL-6, IL-8, MCP-1, MIP-1α, MIP-1β, RANTES, TNF-α, EGF, Eotaxin, FGF-2, GM-CSF, HGF, IL-1β, IL-1RA, IL-15, IL-5, IP-10, I-TAC, MDC, MIF, MIG, VEGF-A. The procedure was as described by the manufacturer’s recommendations. BALF samples were used undiluted, and test plates were run using a Luminex MAGPIX instrument (Luminex Xponent software version 4.2).

### 2.13. Statistical Analysis

Clinical (weight, temperature, heart rate, respiratory rate, spO2) and hematological parameters were evaluated against normal limits. Flowcytometry was performed using a BD FACSymphony instrument, data were collected using BD FACSDiva software, v9.0 (BD Biosciences)) and analyzed using Flowjo. The results were analyzed and graphed using Graphpad Prism 9 software or R. Non-parametric Mann–Whitney U test or two-way Analysis of variance (ANOVA) with Sidak’s multiple comparisons test comparing group means were used to assess statistical significance. The tests used are indicated in figure legends.

## 3. Results

In this study, we tested a previously developed NDV-based vaccine expressing the full-length SARS-CoV-2 spike protein in Cynomolgus macaques [29]. We employed a prime-boost strategy 28 days apart, followed by SARS-CoV-2 challenge, as shown in Figure 1A. Each animal received a total of 5 × 10^7^ PFU of NDV expressing either full-length SARS-CoV-2 spike protein or GFP (NDV-GFP) as a control via a combined intranasal/intratracheal instillation method (2.5 × 10^7^ PFU per route). This instillation method was chosen to allow for penetration of the NDV vaccines into the lungs of the animals, in an attempt to generate a robust immune response within the lungs, similar to what might be achieved through aerosolization.

### 3.1. Pre-Challenge Immune Profile

After prime administration, serological testing showed seroconversion in three of four vaccinated animals by day 14 and in all four by day 28 (Figure 1B). Anti-spike IgG ELISA titers increased following the boost in all animals but were not significantly higher than day 28 titers, while no detectable IgG titers were seen in the NDV-GFP animals (Figure 1B). Using serum collected prior to challenge with SARS-CoV-2, two NDV-FLS animals had detectable anti-spike IgM, while no animals had detectable levels of spike-specific IgA (Figure 1D).

The microneutralization assay showed development of detectable levels of neutralizing antibody titers in the serum of all four vaccinated animals, which appeared in three of four animals only following the administration of the boost (Figure 1C). A single vaccinated animal also saw a two-fold reduction in neutralizing titer just between the final pre-challenge bleed and the bleed performed on the day of challenge. All animals had neutralizing antibodies on the day of challenge, with similar ELISA titers to each other throughout the course of the study.

Bronchoalveolar lavage (BAL) and whole blood samples were collected on days 14 and 28 post-prime, as well as day 16 post-boost to examine the presence of antibodies and specific immune cell populations in circulation and in the lungs of vaccinated animals. None of the control or vaccinated animals had detectable SARS-CoV-2-specific IgM, IgG, or IgA in the BALF. The amounts of CD4 and CD8 T cells in the BALF were not different between animals that received NDV-FLS and NDV-GFP (Figure S1). This is likely due to both groups of animals receiving live viral-vectored vaccines via a mucosal route, thereby triggering an inflammatory response. The immunophenotyping assay used here cannot distinguish antigen-specific immune responses in the cells examined, which are likely present. Further immunological analyses using peptide library analysis are warranted.

### 3.2. SARS-CoV-2 Challenge, Clinical Findings, and Imaging

**Figure 2:**
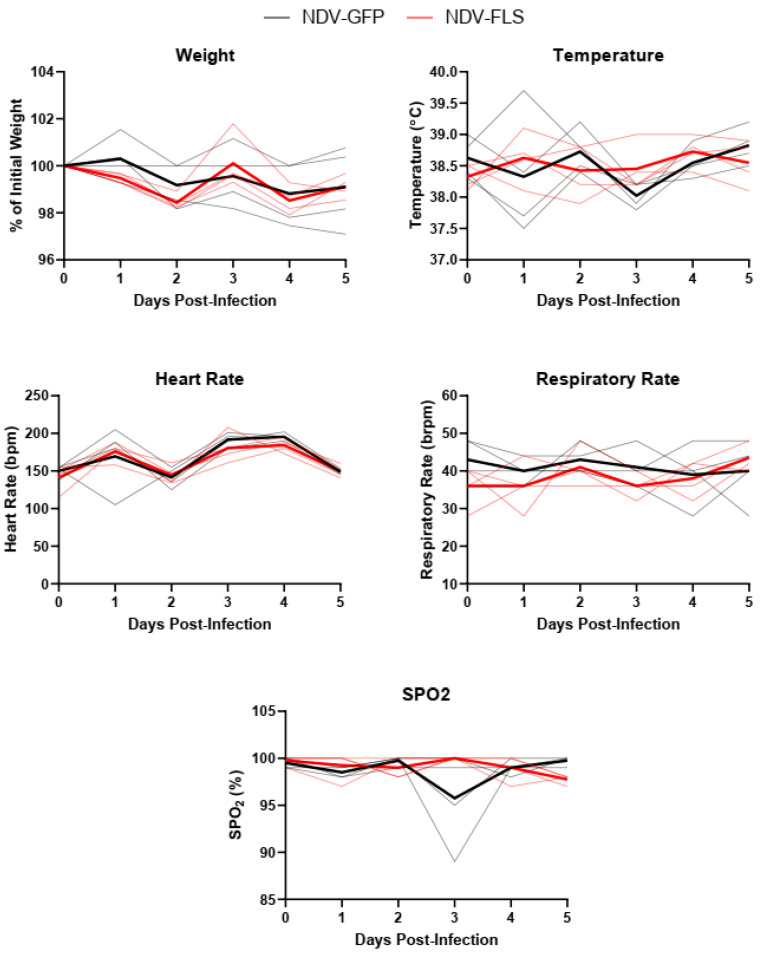
Animal vital signs throughout SARS-CoV-2 infection. Weight, temperature, heart rate, respiratory rate, and peripheral oxygen saturation measured daily following SARS-CoV-2 infection. Solid thick black and red lines represent the mean for each group, while semi-transparent lines represent each individual animal. *n* = 4.

**Figure 3:**
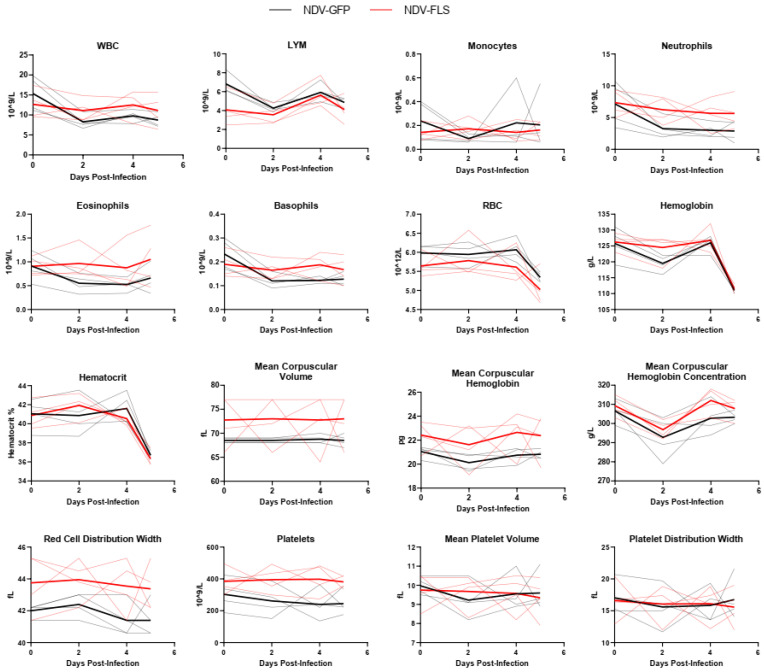
Hematological findings in SARS-CoV-2-infected animals. Complete blood counts and hematological analysis of all infected animals following SARS-CoV-2 infection. Solid thick black and red lines represent the mean for each group, while semi-transparent lines represent each individual animal. *n* = 4.

**Figure 4:**
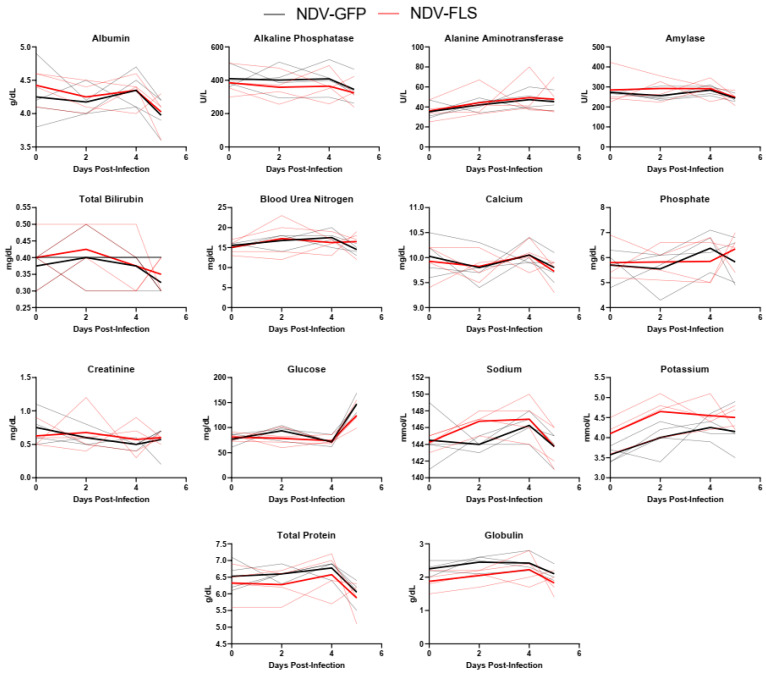
Serum Biochemistry in SARS-CoV-2-infected animals. Serum biochemistry analysis of all animals following SARS-CoV-2 infection. Solid thick black and red lines represent the mean for each group, while semi-transparent lines represent each individual animal. *n* = 4.

Twenty-one days following the second dose of the vaccine, all animals were challenged with a total of 10^6^ PFU of SARS-CoV-2 via a combined intratracheal, intranasal, oral, and intraocular route, similar to that described previously [45]. After inoculation, animals were monitored daily (Figure 2). No significant changes in weight, temperature, heart rate, blood pressure, or peripheral oxygen saturation were seen throughout the course of infection. Only two animals in the control group showed slighter lower oxygen saturation and labored breathing on day three; an additional animal experienced labored breathing on day four post-infection (pi) but not decreased oxygen saturation (Figure 2). Our data align with previous reports of SARS-CoV-2 infection of cynomolgus macaques which indicate that infection causes mild illness with few clinical signs [46,47,48]. Despite some visible signs of labored breathing, no radiographic abnormalities were seen on any day of the experiment (Figure S2). This may reflect a lack of or very mild involvement of the pulmonary parenchyma, which might have been beyond the diagnostic sensitivity of X-rays. Hematological and serum biochemical parameters, which were assessed every day up to day five post-challenge, showed no values outside of the normal range, and no differences between the vaccinated and unvaccinated groups were observed (Figure 3 and Figure 4). This is possibly due to the mild nature of infection resulting in limited variation in these parameters. The clinical course of COVID-19 in humans has been reported to consist of a coagulopathic phase, in which the presence of blood clotting can result in complications [49]. We measured the fibrinogen levels as well as thrombin, prothrombin, and activated partial thromboplastin times in all infected animals throughout infection (Figure S3). While control animals appeared to have a slightly higher mean activated partial thromboplastin time compared with the vaccinated animals on day 2, there were no significant differences between groups, and all values appeared to be within normal limits.

### 3.3. Virus Shedding and Replication in Tissues

**Figure 5:**
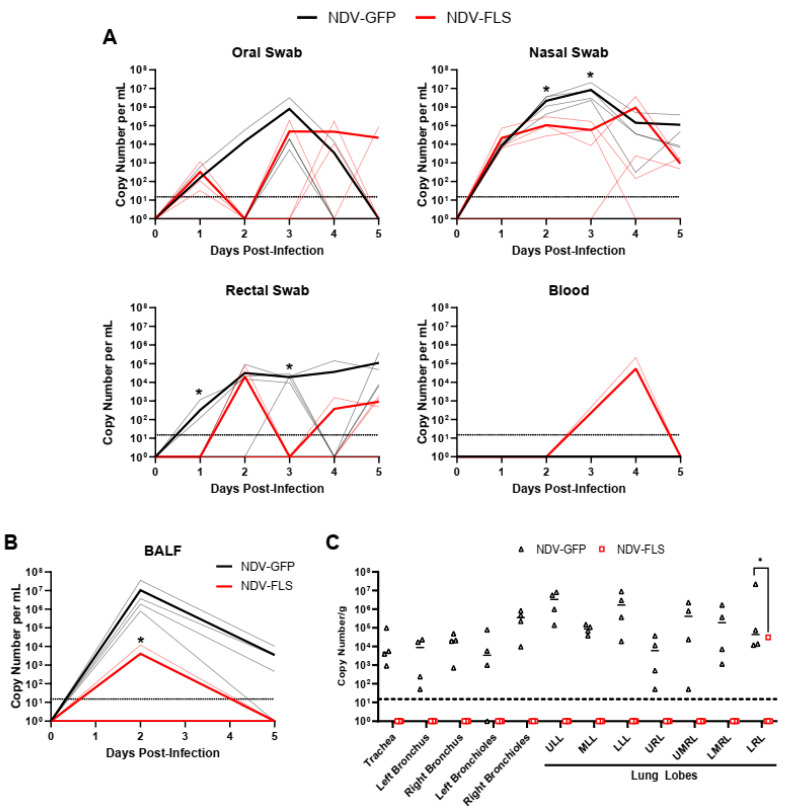
SARS-CoV-2 detection in vaccinated and control animals. Following infection, SARS-CoV-2 RNA copies were determined in (**A**) oral, nasal, and rectal swabs as well as in whole blood. Symbols have been added to oral swab lines for determination of individuals over time. (**B**) SARS-CoV-2 RNA copies detected in BALF on days 2 and 5 pi. (**C**) SARS-CoV-2 RNA copies in respiratory tissues on day 5 pi. Data for each individual animal are shown. Solid thick black and red lines represent the mean for each group, while semi-transparent lines represent each individual animal. *n* = 4. Significance was assessed by Mann–Whitney test in (**A**,**B**) and by two-way ANOVA with Sidak’s multiple comparisons test in (**C**). * *p* < 0.05.

The daily oral, nasal, and rectal swabs collected indicated that the peak of viral replication occurred on day three pi, as measured by the presence of SARS-CoV-2 RNA (Figure 5). Detection of viral RNA in oral swabs was inconsistent across the experiment when examining individuals from each group. Viral RNA was more readily and consistently detected in rectal swabs of NDV-GFP vaccinated animals compared with the NDV-FLS group, which had only one animal with detectable RNA on day 2, one on day 4, and two on day 5 in the vaccinated group (Figure 5A). RNA levels were significantly higher in the NDV-GFP group in rectal swabs on days 1 and 3. Animals vaccinated with NDV-FLS had significantly lower levels of viral RNA in nasal swabs on days two and three pi, during the peak of viral replication (Figure 5A). Mean viral RNA levels were 20.3-fold lower in vaccinated animals on day 2-, and 142-fold lower on day 3 (2.16 × 10^6^ copies vs. 3.6 × 10^5^ copies on day 2 and 8.41 × 10^6^ copies vs. 5.92 × 10^4^ copies on day 3, respectively). Following infection, we also tested the BALF from each animal on days two and five pi for the presence of SARS-CoV-2 RNA. At day two pi, all four control animals had detectable viral RNA in the BALF, while only two vaccinated animals had detectable viral RNA, while mean viral RNA levels were significantly reduced, more than 2500-fold, in vaccinated animals (Figure 5B; 1.05 × 10^7^ copies vs. 4.07 × 10^3^ copies, respectively). On day five, no vaccinated animals had detectable viral RNA, while three control animals still had detectable RNA, though at levels that were reduced compared to day two (Figure 5B). A single animal, in the vaccinated group, had detectable viral RNA in the blood on day four pi (Figure 5A).

The presence of infectious virus was also tested in the nasal swabs and BALF. Three animals in the NDV-GFP group had low but detectable levels of infectious virus in BALF on day 2 (Supplementary Table S1; animals 8158, 6768, and 0673; data not shown). Similarly, only two animals had detectable infectious virus in nasal swabs (8158 on day 2 and 0673 on day 4; data not shown). On day five, all animals were euthanized to determine the extent of the viral burden in the tissues and to examine any gross pathological lesions in the lungs, and to collect tissues for histopathological analysis. RNA was extracted from tissues of the respiratory tract to detect SARS-CoV-2 RNA. Viral RNA was not detected in the respiratory tract of any of the tissues from vaccinated animals (total of 48 samples), with the exception of a single positive sample from the lower right lung lobe of macaque 9670, though viral RNA levels were still significantly reduced in this lung lobe, as assessed by two-way ANOVA (Figure 5C). On the other hand, high viral RNA levels were detectable in all tissues of the control animals (total of 48 samples), with the exception of one sample from the left bronchioles from animal 0673. Interestingly, infectious virus was not detected in any of the tissues examined, even those positive by molecular analysis. This is likely due to euthanasia occurring after the peak of viral replication and as the animals were in the process of clearing the infection. Nevertheless, the drastic differences seen in the proportion of positive animals and magnitude of virus replication, as seen in these samples, indicate that vaccination significantly decreases SARS-CoV-2 replication in macaques, leading to more rapid and efficient clearance of the virus.

### 3.4. Macroscopic Lesions and Histopathology

Similar lesions were noted upon gross examination of the lungs collected from the animals upon necropsy (Figure S4). While mild to moderate inflammation and lesions were noted in some vaccinated animal lung lobes, a wide spectrum of severity in terms of lung lesions were noted in control animals, with some lobes displaying severe pathology.

**Figure 6:**
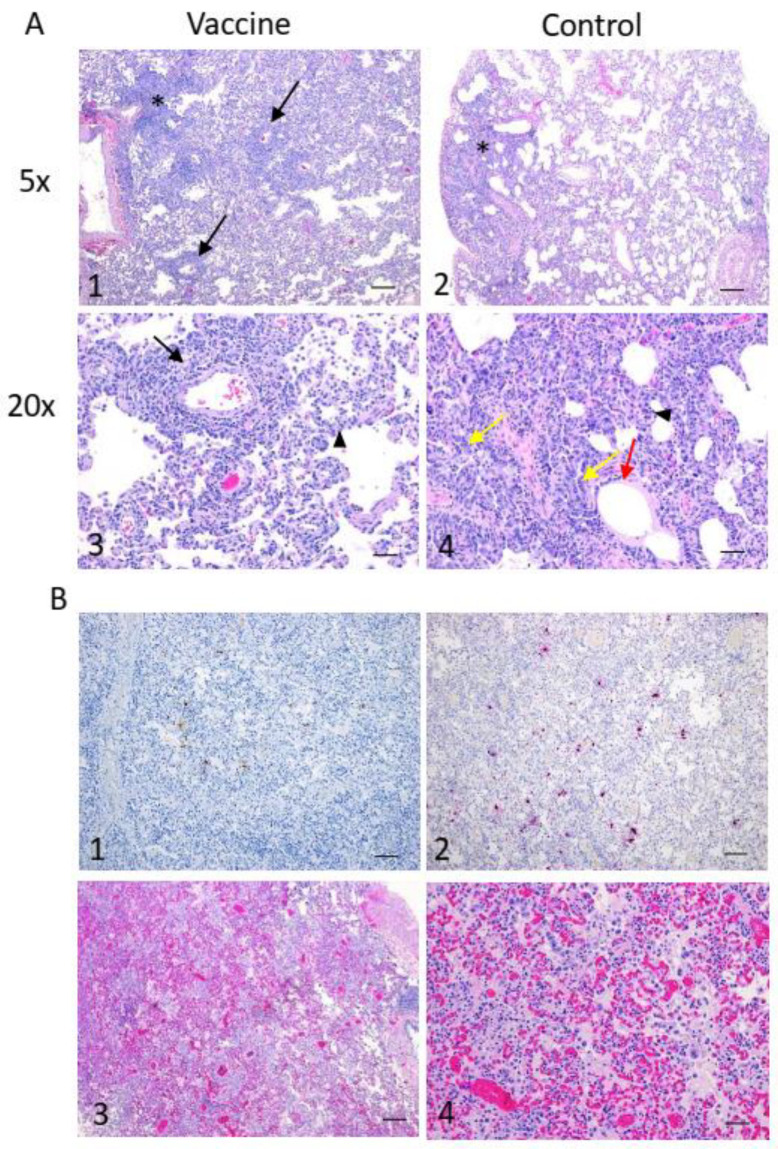
Histopathology, immunohistochemistry, and in situ hybridization. Findings in animals experimentally infected with SARS-CoV-2. (**A**) Similar mild to moderate lesions were observed in both groups and included perivascular inflammation (1, 3 black arrows), multifocal areas of discrete interstitial pneumonia (1, 2, denoted by *) and interstitial infiltrates of inflammatory cells (3, 4 arrowheads). Bronchiolitis (4, red arrow) and type II pneumocyte hyperplasia (4, yellow arrows) were also observed in both groups, however were more prominent in the control group. (**B**) In 2/4 control animals, viral antigen (1, brown) and viral RNA (2, pink) could be detected. Severe lesions were observed in the lower left lobe of one of the control animals and included extensive edema/congestion (3) and alveolitis (4). Bar = 200 µm for A1, A2 and B3, all other panels bar = 50 µm.

Mild to moderate lung lesions were observed in both the NDV-GFP and NDV-FLS groups and there was no significant difference in average lesion scores between the 2 groups (Figure 6) (control = 10.7, vaccinated = 12.0). The lesions observed often included perivascular inflammation, multifocal areas of discrete nodular interstitial pneumonia, interstitial infiltrates of inflammatory cells, bronchiolitis, and type II pneumocyte hyperplasia (Figure 6A).

Using immunohistochemistry and in situ hybridization, viral antigen and RNA, respectively, could be detected in 2/4 NDV-GFP animals (Figure 6B) and no NDV-FLS-vaccinated macaques. In one of these NDV-GFP-vaccinated animals, severe lesions were observed in the lower left lobe and included extensive edema, congestion, alveolitis, and intra-alveolar multinucleated and atypical cells in addition to the more frequently observed inflammatory indicators previously listed. Overall, while all animals showed some signs of pathology following infection, a lack of viral antigen and RNA supports the RT-qPCR data and suggests that NDV-FLS-vaccinated animals were able to clear the virus readily following infection, compared to controls.

### 3.5. Inflammatory Response Analysis

Following infection, we performed a multiplex Luminex assay to examine the levels of multiple cytokines and chemokines in the BALF of infected animals. While the majority of cytokines were at or below the limit of detection of the assay, and many had changes that were unremarkable between groups and across time points, some were elevated following SARS-CoV-2 infection. Three of four NDV-GFP-treated animals had increased Interferon-inducible T-cell alpha chemoattractant (I-TAC) and Interferon gamma-induced protein 10 (IP-10) in the BALF on day 2, which then fell to baseline levels by day 5 (Figure S5). At day 2, 3/4 and 2/4 NDV-GFP-treated animals had increased levels of IL-1RA and IL-6, respectively.

## 4. Discussion

The last four years of the COVID-19 pandemic saw extraordinary amounts of morbidity and mortality worldwide. Since 2021, SARS-CoV-2 variants that have mutations in key residues that are important for spike protein binding to ACE-2 have emerged independently in multiple countries and these variants have been in circulation since [50,51]. Reportedly, the increased transmissibility of these variants as well as their potential for escaping immunity induced via infection or vaccination make advancements in vaccine technology and development of critical importance. Despite the record-setting development of SARS-CoV-2 vaccines and the rapid nature of their deployment in the first year of the pandemic, there remains a need for next-generation vaccine candidates to continue through pre-clinical and clinical development stages. Supplies of currently approved vaccines cannot meet global demand, highlighting the need for additional candidates. The use of different vaccine platforms provides diverse options, and in the event of future pandemics, prior knowledge of the efficacy of given platforms and the ability to deploy these platforms quickly will be highly sought after. Therefore, the ongoing production and testing of various vaccine platforms against SARS-CoV-2 is critical not only for new vaccines against SARS-CoV-2 but also for informing our response to future emerging viral outbreaks and possible pandemics. To this end, we tested an NDV-vectored vaccine candidate, previously shown to be highly efficacious in a Syrian hamster model, in a non-human primate model of SARS-CoV-2 infection [29].

Previous reports of SARS-CoV-2 infection in cynomolgus macaques have shown that they are susceptible to infection and show signs of mild disease, including inflammation and diffuse alveolar damage in the lungs [46]. This model has also been used to examine the efficacy of hydroxychloroquine against SARS-CoV-2 infection [48]. We vaccinated four cynomolgus macaques with NDV-FLS via a mucosal/respiratory combined intranasal and intratracheal route. This route has been shown to be effective with NDV-vectored vaccines previously, and, while it is not currently adopted in clinical practice, it appropriately primes an immune response in parts of the body that are commonly targeted by the virus [29]. Two doses of NDV-FLS induced anti-spike IgG responses in all animals, a response that increased in magnitude after the second dose but did not result in significantly higher antibody titers. Neutralizing antibody responses were detectable in all animals after two doses. The levels of neutralizing titers were comparable to those seen in hamsters administered the same vaccine. Vaccination (prime only) with NDV-FLS also was able to provide significant protection in hamsters infected with SARS-CoV-2 in the absence of neutralizing antibodies, suggesting that serum neutralizing activity may not be critical for protection in other models, and their importance here should be assessed further. Others have reported varying levels of neutralizing antibodies across a wide range of species, dependent on the route of immunization [40,41,42,43]. A recent human trial showed that vaccination with an NDV-based vaccine resulted in a higher proportion of neutralizing antibodies within the antibody repertoire compared with mRNA-based vaccination [52]. It is noteworthy that the vaccine used here contained wild-type SARS-CoV-2 spike protein, while other versions and next-generation vaccines have included pre-fusion stabilized spike protein, which can influence the generation of neutralizing antibodies. Interestingly, vaccinated animals did not have detectable SARS-CoV-2 spike-specific IgA responses in either the serum or BALF (Figure 1D). Undetectable IgA levels in serum may be due to low levels of IgA being induced generally. The lack of IgA detection in BALF is likely due to a low concentration of IgA, if present, in the BALF. The procedure results in a large volume of aspirated PBS (20–30 mL), which significantly dilutes the presence of antibodies and other proteins and analytes being examined. In future studies, concentrating the BALF to enable detection of antibodies could be considered, along with testing for IgA in lung tissue homogenates. We did not examine immunity to any variants of concern in this study as it was carried out prior to their emergence. However, this will be an area of interest given that Omicron lineages currently dominate globally and are antigenically very distant from ancestral Wuhan SARS-CoV-2. Either developing newly matched NDV vaccines to circulating viruses as needed, depending on immune escape and predominance, or assessing how well expression of earlier spike proteins can protect against recent variants will be a priority moving forward with the platform. We have shown evidence that protection against matched strains is likely to be provided, and there is strong evidence that even less closely matched vaccines can provide some degree of protection even with modest neutralization, as seen here, likely through non-neutralizing mechanisms or cell-mediated immunity. Overall, the adaptability of the NDV platform would allow for the generation of new vaccines seasonally, akin to the development of flu vaccines, given their similar production pipelines, and updating vaccines for human use would likely be practical and feasible.

Following two vaccine doses, each animal was infected with 10^6^ PFU of SARS-CoV-2 and monitored for five days throughout the course of acute infection. Throughout the course of the experiment, no animals suffered from severe clinical signs, and all vital signs and measured clinical parameters remained normal throughout the experiment, with the exception of two control animals that had reduced oxygen saturation on day 3 pi. This mild clinical course was expected and similar to previous reports and coincided with the peak of viral replication between 2 and 3 days pi. Pathological analysis showed only mild lesions in both vaccinated and control animals. Similar gross and microscopic pathology was noted in both vaccinated and control animals. Similar lesion development in both vaccinated and control animals could also be due, in part, to lesions developed following vaccination with NDV vectors, as both control and vaccine groups received live NDV. However, tissue collections were performed 26 days following the last NDV dose and after SARS-CoV-2 infection, making inflammatory responses to infection a more likely cause of the pathology seen. Overall, the low severity of microscopic lesions might have been caused by the fact that necropsy was carried out at day 5 pi, which was slightly after the peak of virus replication seen in nasal swabs. This time point was chosen to allow for the development of any clinical signs and histopathological lesions, which were previously shown to be present on day 4 pi in this animal model [46].

Significant reductions in viral RNA levels were seen in the respiratory tract of NDV-FLS-vaccinated animals compared with controls (Figure 5). The lack of viral RNA in the respiratory tissues of vaccinated animals suggests that immunity in both the upper and lower airway was able to efficiently clear the virus upon infection. This is a critical step in reducing or eliminating disease development. Interestingly, we did not detect infectious virus in the tissues collected upon necropsy on day 5 pi. This is likely due to the period of acute infection occurring days earlier, with the amount of infectious virus being markedly reduced already at the time of necropsy. The reduction in viral RNA levels in vaccinated animals was also seen in the BALF and in nasal and rectal swabs, suggesting that vaccination may limit the amount of viral shedding in infected individuals, a critical aspect for a successful vaccine. While we did see reductions in shed viral RNA, further experiments into the efficacy of the vaccine in preventing or reducing transmission, besides abating clinical signs and lesions, are needed and are of high interest. As many of the current vaccines are administered intramuscularly, the development of mucosal immunity in the airways could be limited, decreasing the ability of current vaccines to effectively curtail infection and transmission. NDV vaccination via the mucosal routes, as described in this study, may provide protection against replication in the nasopharynx, effectively decreasing viral transmission. While we could not detect SARS-CoV-2-specific antibodies in the BALF, vaccination decreased virus replication in lung tissues, as well as virus RNA in the BALF and swabs, suggesting that BAL immunoglobulin may not be the only correlate of protection regarding mucosal immunity.

A wide variety of clinical biomarkers have been identified that may inform clinical prognosis or be indicators of severe disease in human COVID-19 cases [53]. We examined a number of hematological and biochemical parameters in infected NHPs and examined each animal’s ability to efficiently form blood clots, as it is becoming increasingly clear that COVID-19 disease progression consists of a coagulopathic stage of disease [49]. Interestingly, most markers did not differ significantly between groups and there were few signs of hematological, kidney, or liver dysfunction as assessed through serum protein and ion levels (Figure 3, Figure 4 and Figure 5). The mild nature of the disease in this model as well as the early time points following infection may account for the lack of perturbations in these factors. Similar findings were seen when the same vaccine was tested in a Syrian hamster model of infection, a model that represents a more severe clinical course, but still does not result in severe morbidity or lethal disease.

While there has been significant progress made in the development and distribution of vaccines against SARS-CoV-2 since the pandemic began, there is still a critical need for continued development and testing of alternative vaccine candidates for COVID-19. This development is important for establishing a number of vaccine candidate options that can be developed quickly and efficiently in the event of future emerging viral outbreaks. Our previous data, as well as our findings here, indicated that the NDV platform is a viable option for further clinical development. This platform’s previous safety profile as well as its ability to provide protection in several models of disease highlight its potential as a breakthrough vaccine candidate. Indeed, several other groups have published pre-clinical studies reporting the efficacy of NDV-based vaccines in a number of models, including mice, hamsters, and immunogenicity and safety in rats and pigs [40,41,42,43,54,55]. Further, early-stage clinical trials were carried out with both inactivated and live NDV-based vaccines in Mexico, Thailand, and Vietnam [36,37,38,39]. These early trials have shown good safety and efficacy profiles, including comparable immunogenicity to mRNA-based vaccination in humans [52]. Despite these advantages, certain questions remain regarding the possible development timeline and future studies should examine the potential impact of anti-NDV immunity on subsequent doses of vaccine or with different NDV-based vaccines following an initial vaccination regimen. Here, we show that our NDV vaccine expressing SARS-CoV-2 spike protein provides protection in a non-human primate model of infection, including the likely elimination of the virus in the upper airway, which should result in a significant reduction in transmission from infected individuals. This may be in part due to the mucosal delivery of the vaccine, which is in contrast with the intramuscular nature of many currently offered vaccines that have received emergency use approval. This route of immunization may provide an additional layer of community protection from infection and disease and is an important aspect of this vaccine’s efficacy profile. We have provided evidence that this vaccine provides an effective alternative to currently available vaccine candidates and warrants further development.

## 5. Conclusions

We report the efficacy of a Newcastle disease virus-vectored vaccine candidate that expresses full-length SARS-CoV-2 spike protein administered to cynomolgus macaques. Animals given two doses of the vaccine via respiratory (intranasal/intratracheal) immunization developed robust immune responses and had significantly reduced viral RNA levels in nasal swabs, BAL fluid, rectal swabs, and lower airway tissues. Our data indicate that NDV-FLS administered mucosally provides significant protection against SARS-CoV-2 infection, resulting in reduced viral burden, and should be considered as a viable candidate for clinical development.

## Data Availability

The raw data supporting the conclusions of this article will be made available by the authors on request. Inquiries should be sent to bryce.warner@phac-aspc.gc.ca.

## Supplementary Materials

The following supporting information can be downloaded at: https://www.mdpi.com/article/10.3390/vaccines12040404/s1, Figure S1: Flowcytometry gating strategy and immunophenotyping of T cells in BAL Fluid; Figure S2: X-rays of SARS-CoV-2-infected animals; Figure S3: Coagulation parameters in SARS-CoV-2-infected animals; Figure S4: Lung Gross pathology of SARS-CoV-2-infected animals; Figure S5: Cytokine and chemokine levels in BAL fluid; Table S1: Animal ID and Group Information.
