# Supplementary material for: Mucosal Vaccination with a Newcastle Disease Virus-Vectored Vaccine Reduces Viral Loads in SARS-CoV-2-Infected Cynomolgus Macaques
Source: Vaccines (Basel). 2024 Apr 10;12(4):404. doi: 10.3390/vaccines12040404 (PMC11054841; doi:10.3390/vaccines12040404)
Supplement: Supplementary file 1 [file vaccines-12-00404-s001.zip › vaccines-2920702-supplementary.pdf]

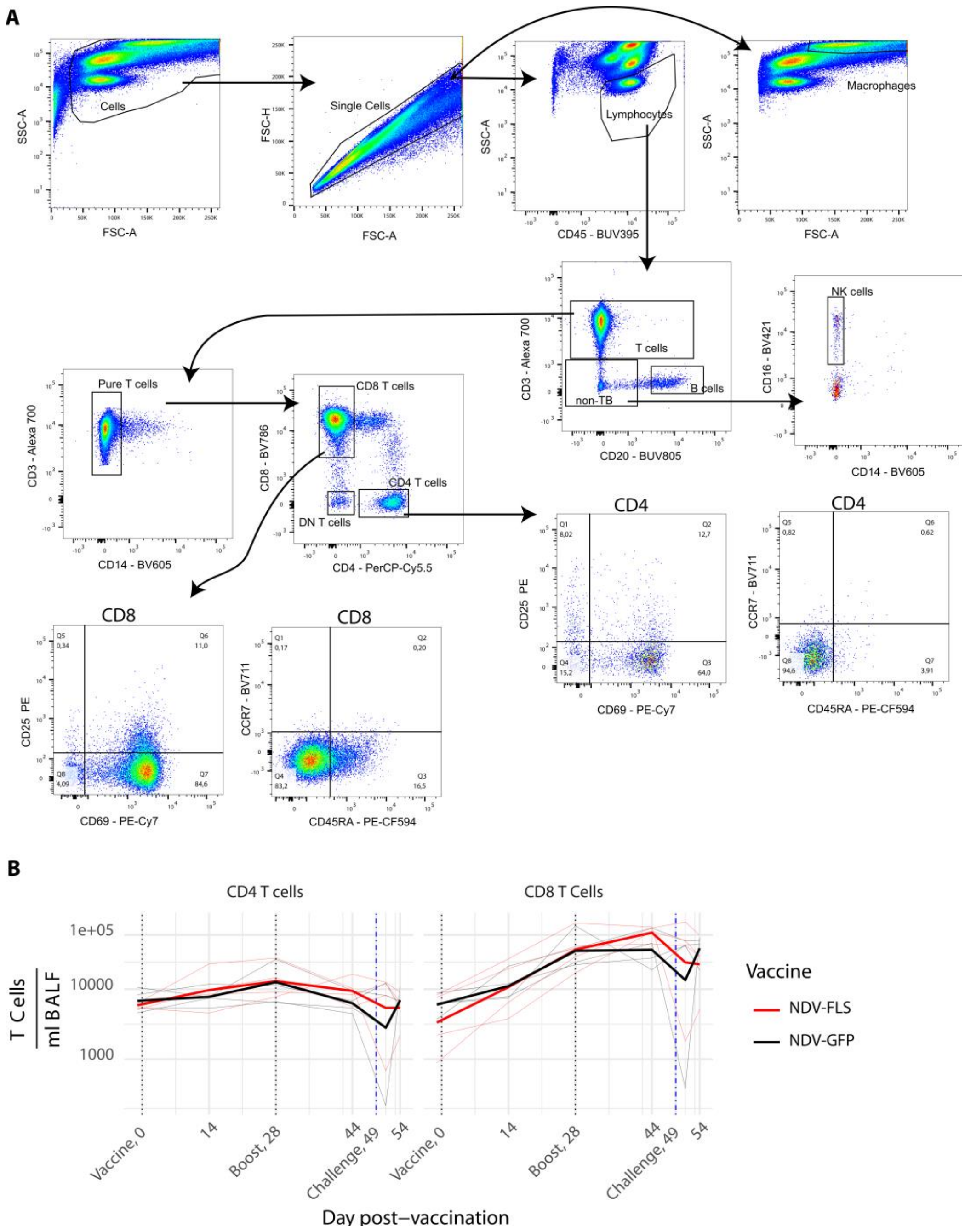

Figure S1. Flowcytometry gating strategy and immunophenotyping of T cells in BAL Fluid. (A) Gating strategy for flowcytometric analysis of T cells present in the BALF. (B) CD4 and CD8 T cells present in the BALF of control and vaccinated animals. Solid thick black and red lines represent the mean for each group, while semi-transparent lines represent each individual animal. n=4.

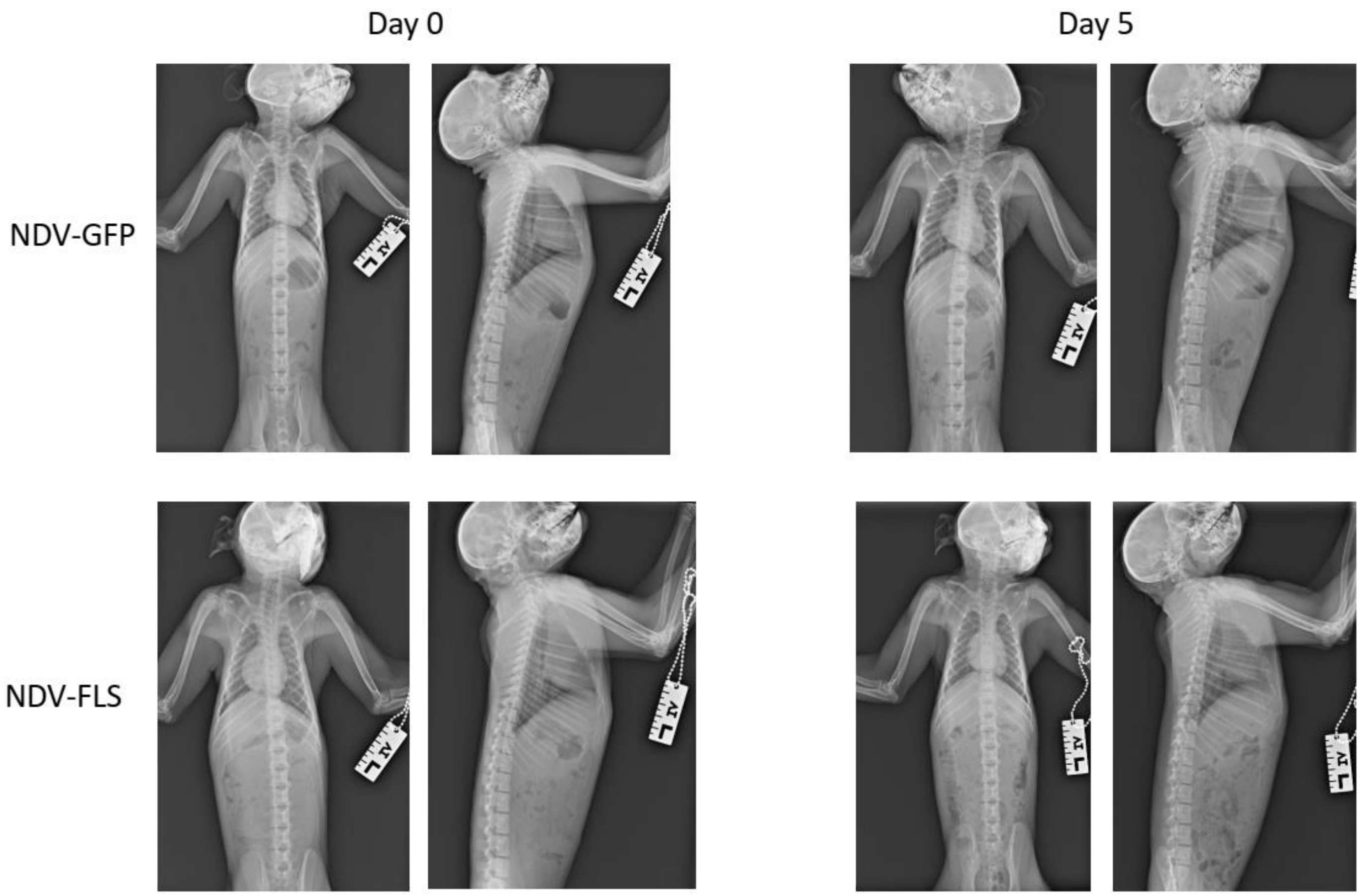

Figure S2. X-Rays of SARS-CoV-2 infected animals. Representative images of x-rays taken on day 0 and day 5 pi of control and vaccinated macaques.

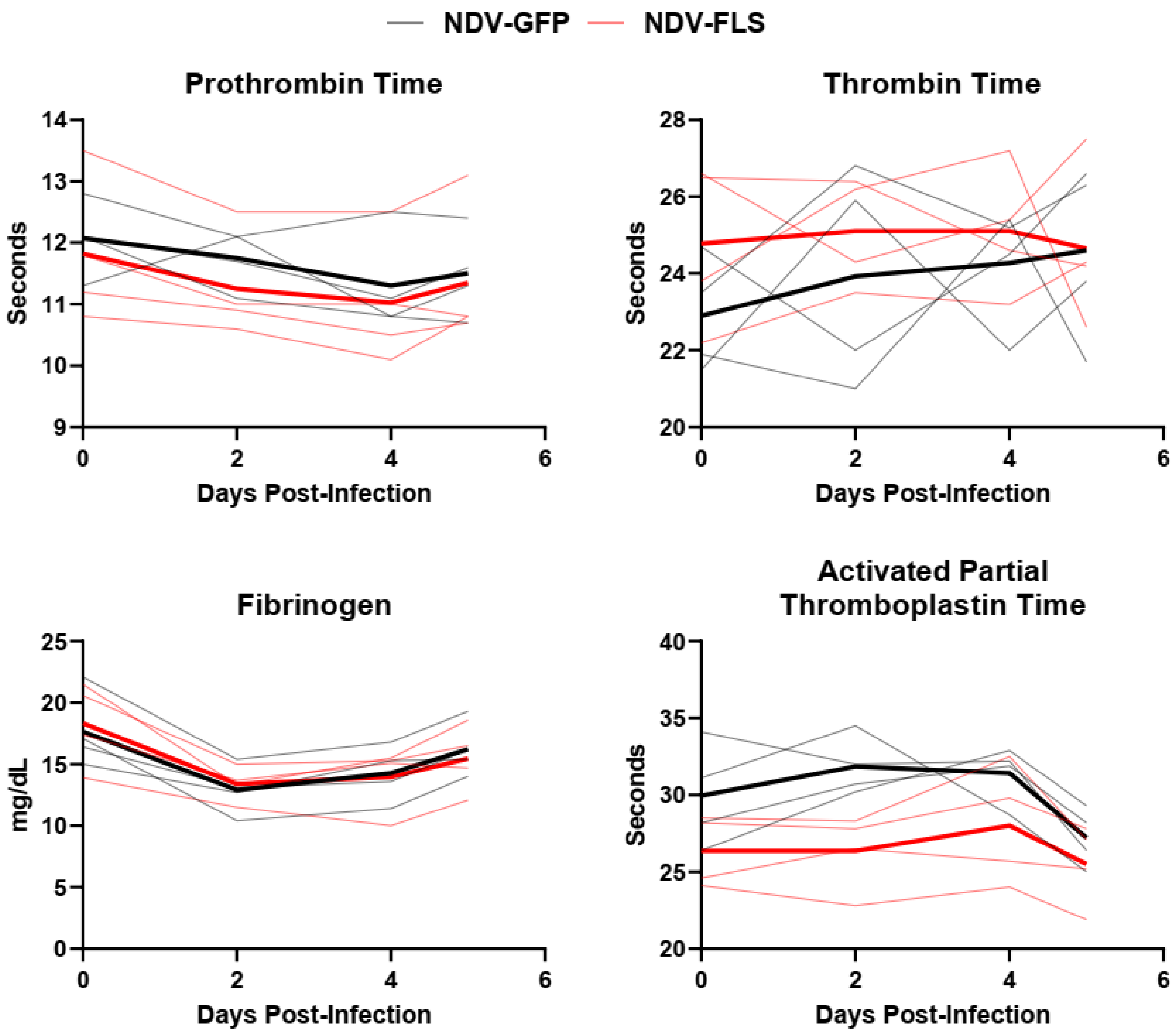

Figure S3. Coagulation parameters in SARS-CoV-2 infected animals. Prothrombin time, thrombin time, fibrinogen levels, and activated partial thromboplastin time throughout infection for all animals. Solid thick black and red lines represent the mean for each group, while semi-transparent lines represent each individual animal. n=4.

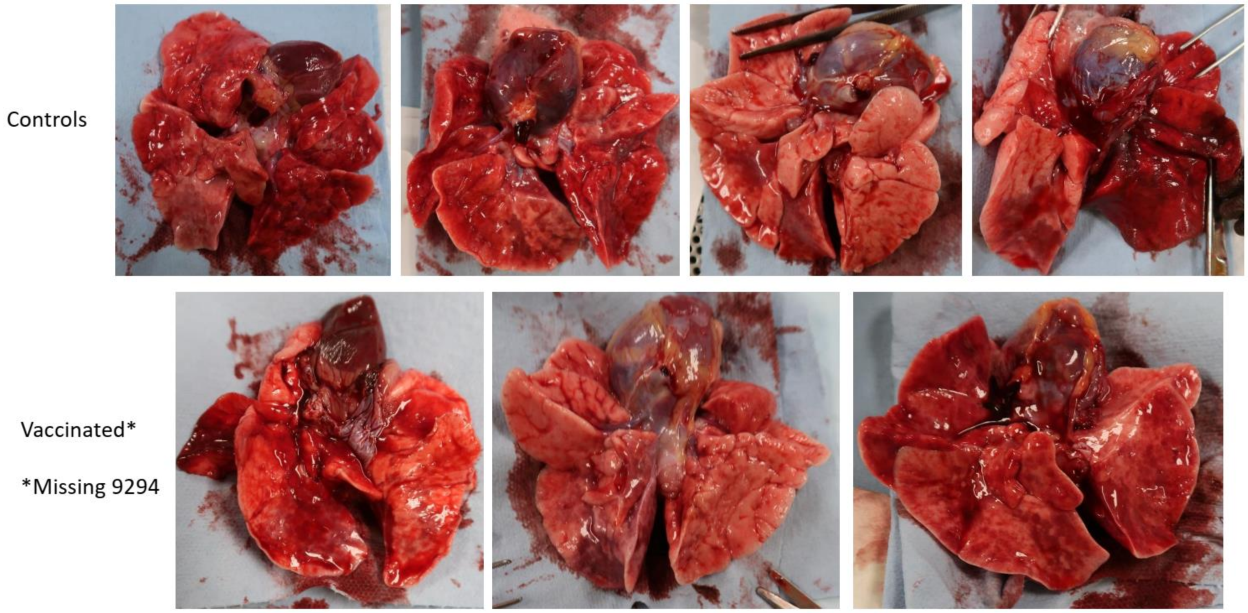

Figure S4. Lung Gross pathology of SARS-CoV-2 infected animals. Images of the lungs of infected animals upon necropsy on day 5 pi. Missing is the image for one vaccinated animal that was not taken (female; animal 9294).

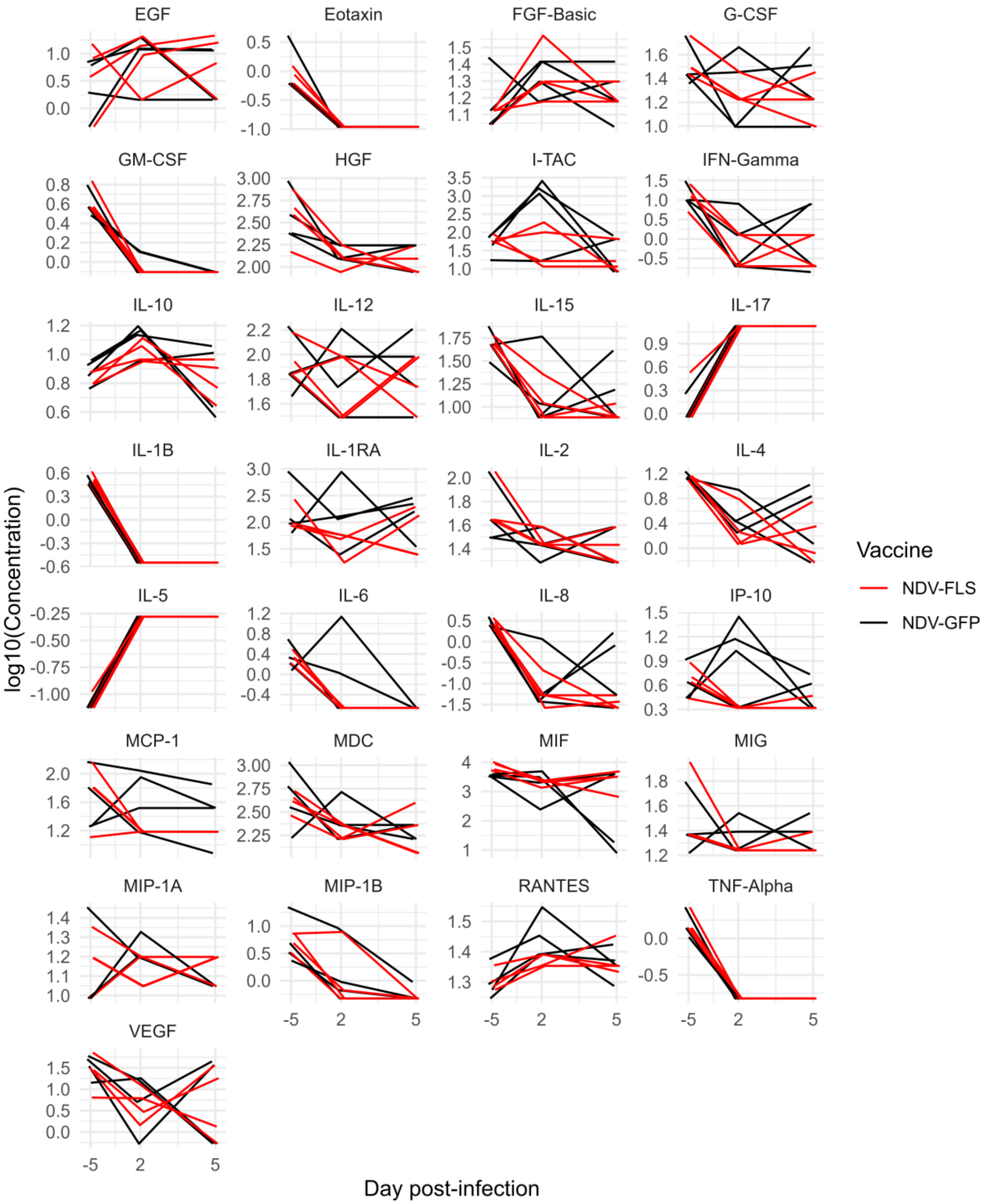

Figure S5. Cytokine and chemokine levels in BAL fluid. Expression levels of the indicated cytokine or chemokine in the BAL fluid of NHP on day 44 after vaccination (5 days prior to challenge) and on days 2 and 5 post-challenge with SARS-CoV-2 were detected by Luminex assay. Data for individual animals are shown at each time point.

Table S1. Animal ID and Group Information.

Animal ID and Sex	Vaccine
6768 M	NDV-GFP
3760 M	NDV-GFP
0673 F	NDV-GFP
8158 F	NDV-GFP
7550 M	NDV-FLS
9670 M	NDV-FLS
8761 F	NDV-FLS
9294 F	NDV-FLS
